# Communication and empathy in an emergency setting involving persons in crisis

**DOI:** 10.1186/1757-7241-16-5

**Published:** 2008-07-27

**Authors:** Halvor Nordby, Øyvind Nøhr

**Affiliations:** 1 Lillehammer University College, Faculty of health and social work, 2604, Lillehammer, Norway; 2 The University of Oslo, Institute of health management and health economics, P.O. Box 1089, Blindern, 0317, Oslo, Norway

## Abstract

The article presents a study of the interaction between paramedics and parents in cases of Sudden Infant Death Syndrome (SIDS). We have sought to understand how the parents perceived the paramedics ability to communicate as well as empathise and deal with practical aspects of the situation. We have also sought to understand how the paramedics view their role as professional health workers, and how they think they should interact with persons in crisis. The method used in this study is qualitative and involves semi-structured interview schemes. We conducted twelve interviews – six with parents and six with paramedics. One of our primary findings is that many of the parents interviewed were not satisfied with the paramedics' communication, empathy and ability to take care of the practical aspects of the situation. The interviews have also revealed that there is significant disagreement among paramedics about the interpersonal role of health workers in situations involving people in crisis. The final part of this article includes a discussion of these and other findings. We argue that guidelines that specify threshold conditions for communication and care should be implemented in education and training. The aim of such guidelines should be to make sure that parents of lifeless children are secured a minimum of relevant explanations, information and care.

## 1. Background

This article presents results from a qualitative research project designed to study and understand interpersonal relations in an emergency setting involving persons in crisis. The study has focused on the interaction between paramedics and parents of children whose deaths were later attributed to Sudden Infant Death Syndrome (SIDS). This interaction is very dramatic and difficult. The paramedics normally encounter the parents with the knowledge that they have a lifeless child, and they also know that the parents are undergoing extreme emotional and cognitive stress. Before the arrival of the paramedics and medical doctors, the parents' only contact with the health system is interactive, by way of medical emergency telephone.

The aim of our research has been to understand how challenges related to communication, care and empathy are experienced in the interaction between paramedics and parents. The next section clarifies this aim of the project and its wider significance to studies of communication in emergency situations. Section three presents relevant theoretical assumptions and the qualitative method used in the semi-structured interviews with paramedics and parents. Section four presents the main results of the study, while the fifth and final section discusses implications of these results.

## 2. Background and study design

Discussions of challenges in health personnel-patient-interaction in emergency situations often focus on the medical aspects of disease or injury [1,2]. This is understandable, since improving health and saving lives are the primary aims of the health services, and since skilled medical treatment is necessary for achieving these aims. It is nevertheless important to remember that emergency situations involve interpersonal relations, and that successful interaction between emergency personnel and their patients often depends on adequate communication [3,4]. Good communication in emergency situations is important for two kinds of reasons. Firstly, communication is a means of avoiding excessive physiological strain and stress that can contribute to a worsening of a patient's condition. It is also important to understand patients' verbal behaviour as clues to their physiological, medical conditions. Secondly, securing good communication is an aim in itself. From the perspectives of patients, the atmosphere created by paramedics' verbal and non-verbal communicative actions often means a lot.

Securing successful communication can prove a formidable challenge, and communicative challenges can be especially acute when time constraints and limited resources are not the only obstacles. In many emergency situations patients or relatives of patients experience physical states such as severe pain and shock and mental states like despair and anxiety. This typically makes it more difficult to secure good communication. Furthermore, the fact that it is difficult to communicate due to a patient's mental and physical experiences of crisis and ill-health does not mean that it is unimportant to communicate. On the contrary, securing adequate communication and care is of special significance when patients experience heavy physiological and psychological strain [1].

The starting point for our study was an interest in understanding how emergency health personnel and persons in crisis experience challenges related to communication, care and empathy in an emergency situation. We did this by focusing on cases of interaction between paramedics and parents whose children were later diagnosed with SIDS. Here we define the term 'paramedic' as health personnel working in the ambulance services and who have a competence that is equivalent to the further national education course 'Nasjonal Paramedic Utdanning' in Norway. There are other ways of understanding the term, but the important point is that our definition is reasonably clear and useful for our purposes.

The parents that paramedics encounter in cases of SIDS are in extreme emotional and cognitive states of shock, despair and disbelief. As one of our respondents characteristically said, 'I was not at all capable of rational reasoning'. Being the first health professionals on the scene, the paramedics' primary focus is on the lifeless child and attempts at resuscitation. However, when time and resources permit, the paramedics need to interact substantially with the parents. It is important that they are given relevant information and taken as good care of as possible.

It is this dimension of the interaction our study focused on. The main purpose of the study was to understand how relevant communicative challenges were perceived by both parents and paramedics. We wanted to understand how the parents experienced the paramedics' communicative actions, empathy and ability to take care of practical aspects of the situation. We also wanted to understand how the paramedics experienced relevant communicative challenges in the dramatic situations included in the study, and how they perceived their role as professional health workers in emergency situations.

It should be emphasised that when we here talk about 'experiences', we were not merely concerned with beliefs and thoughts that the parents and paramedics formed in the course of interaction. Our focus also included emotions, sense impressions and other psychological states related to the events experienced. Furthermore, we were not merely concerned with experiences that were regarded as salient in the moment of the crisis. The reason for this is obvious: events that were not regarded as important at the time of the interaction could later be regarded as extremely important. We were, for the same kind of reason, not concerned merely with what the parents thought of as 'good' actions. If we had focused too much on evaluative judgements, we would have risked excluding something important [5,6]. By adopting a holistic focus our goal was to reveal all the aspects of the interaction that were important to our research questions.

## 3. Method

It was important to communicate face-to-face with our respondents in an atmosphere that felt safe and comfortable for all parties involved. The interviews involved a significant strain for the respondents, especially the parents. It was of vital importance for us, as researchers, to acknowledge their difficulty. There were a number of consequences that needed to be avoided. These consequences not only concerned the psychology and experiences of the parents and the paramedics, but also the problematic assumption that it is possible to uncover objective truths about interpersonal relations and communicative processes within the scope of an interview [7,8].

The aim of the project has been descriptive – we have not attempted to evaluate the stories that have been told. It has therefore been important for us to let the participants' own voices be heard. This does not mean that we assume that there are objective interpretations, and that our interpretations of our respondents' stories are neutral and not coloured by theory. The point is that it is always possible to talk about degrees of objectivity. We have attempted to avoid interpretations that are grounded in idiosyncratic subjective horizons or abstract and often controversial theoretical frameworks [7,9,10].

The idea that the aim of understanding is a process that should be as theory-neutral as possible belongs to what Gubrium and Holstein [8] call a 'naturalistic perspective' within qualitative research. Contrary to other perspectives that attempt to determine underlying truths or psychological explanatory mechanisms, a naturalistic perspective seeks to identify a respondent's point of view without interpreting verbal and non-verbal actions on the basis of theories about psychology, truth or knowledge. Philosophical concepts like 'narrative', 'life world' and 'phenomenology' are central to a naturalistic perspective on qualitative interviews [8,11].

It is a widely held view that processes of communication and interpretation do not involve the use of traditional natural science methods. This view has led some researchers to question whether qualitative methods and hermeneutical analyses of understanding are sufficiently scientific [12,13]. It would fall outside our focus to discuss this methodological question. The important point here is that it seems *prima facie *correct to use face-to-face in-depth interviews in a project like ours, and thus our aim has been to make reasonable analyses of the recorded dialogue with our respondents.

The interview scheme that we used in order to overcome the challenges implicit in a project such as this is often called 'semi-structured' [14]. We designed a thematic plan for how the interviews should be conducted – an interview guide – but this plan could be modified according to the circumstances of the interviews. This typically happened when respondents talked about an aspect of the interaction that seemed important for our research but nevertheless not covered in the original thematic plan. Other cases included situations in which the respondents initiated dialogue about an issue we would have returned to later in the plan. In such cases it was often natural to discuss the issue right away, instead of creating an artificial disruption or change of topic.

## 4. Participants

Finding participants for a project like this was a challenging task. Quite a lot of energy went into finding respondents, especially when we tried to find parents who were willing to tell their stories. One obvious reason is that there are not many cases of SIDS in Norway. Another reason is that it is natural to assume that not all parents who have experienced this kind of interaction want to participate in research projects like this. We met these challenges by using 'Landsforeningen uventet barnedød'  and its journal to advertise for volunteers. Those who were interested answered our invitations, and we chose randomly six pairs of parents for interviews. The only requirement for inclusion was the loss of a child from SIDS. We did not pay attention to age, time lapsed since they lost their child, or any other variables when choosing our subjects.

We encountered similar challenges when we attempted to find paramedics. It was difficult to send the research study invitation to all of the paramedics working in the health services in Norway. We solved this problem by using an internet site used by very many paramedics in Norway . We received many responses to our invitation and randomly chose six respondents. Again, we used no other criteria other than the requirement that the volunteers had experienced one or several cases of SIDS.

Some may perhaps suggest that the best arrangement would involve interviews with the parents and paramedics who actually met each other. For practical reasons this was impossible to achieve. It was not probable that all the paramedics who the parents met would want to participate in the study. Moreover, we are not confident that an arrangement of this kind would improve the quality of the research. Our aim has not been to understand each situation and determine who had the 'correct' understanding and who did the 'correct' things. Our aim instead has been to say something general about encounters between paramedics and parents in the hectic and dramatic circumstances on which we have chosen to focus. A discussion and comparison of different interpretations of particular situations would easily fall outside this focus.

We think that we have managed to find a good selection of respondents, and we are satisfied with the thematic dimension of our interviews. By choosing not more than twelve respondents we were able to make comprehensive in-depth interviews, which gave us rich knowledge of each particular situation. Twelve different stories gave us at the same time a sound qualitative basis for understanding how the interaction between the paramedics and parents typically was experienced.

All of our informants clearly and coherently described how they experienced the encounters during the time of crisis. The parents told us many different stories, and the interviews have revealed how paramedics think about their roles as caregivers. In the subsequent sections we present and discuss our main findings.

## 5. Results

All of the parents reported some degree of poor communication. Some parents were reasonably satisfied with the explanations and information provided by the paramedics, but maintained that communication was not optimal throughout the encounter. A typical view was: 'They [the paramedics] could have explained better what they were doing [when the paramedics performed resuscitation].'

According to most of the parents, the problem was not that the paramedics used an unfamiliar, technical language or failed to secure their attention. The paramedics were good at using a language that the parents understood, and the paramedics' communicative style – the way in which they addressed the parents – was also felt to be appropriate by the parents. The problem, many of the parents expressed, was that the paramedics did not communicate all relevant information.

This finding may be attributed to the fact that the paramedics were performing resuscitation and did not have time to address the parents. However, several parents thought that the paramedics could have explained the situation more fully than what they did. This was most obvious in cases in which many paramedics were present. A typical statement made by one of the interviewed parents was as follows: 'I think there were seven persons in our house, but no one told me exactly what they were doing to our child.'

The parents' reports of insufficient explanations were not always presented as a strong criticism. Several parents emphasized that they understood that it was difficult for the paramedics to communicate since their primary focus was on the lifeless child. Other parents were more critical and held that the paramedics did not explain as much as it was reasonable to expect. As one mother said, 'It should not have been too difficult to explain some of the things they did'.

An interesting finding is that most of the parents were not so critical of the paramedics' ability to show care and empathy. Several parents made it clear that they did not have very high expectations about this. As one parent said, 'In the health services today, health workers are not so much concerned with care'. All of the families, however, agreed that empathy is an attitude and skill that paramedics ideally should have. One parent noted that 'We understand that it can be difficult, but it takes so little to show a minimum of care that can mean so much.'

Some families experienced empathy that they thought met this condition. This was empathy that they described as good or at least reasonably good. A striking aspect of our interviews was that the parents remembered instances of empathy very well. One mother told us about the transportation to the hospital, that she felt she was 'placed on the sideline' and received little empathy and care. Among other things, she was asked to hold her child, even though she wanted the paramedics 'to have my child since they were in the best position to take care of her'. However, at one moment she felt that the 'distance between herself and the paramedics was greatly reduced', when 'one of them gave me a wink and an encouraging comment'. This wink and the attention she received were very important to her.

Another parent experienced the following episode:

*I just had to go outside, I had to get some fresh air...I remember he followed me, but kept his distance. So I said: I probably shouldn't ask if you've got a cigarette? I was given one, and I don't even smoke normally...He stayed with me all the time and talked to me, I've no idea what about. I replied, but can't remember what we talked about. I think we talked about things in general and also what they were doing in the ambulance. A few time he tried to explain what was happening, but he probably understood that it didn't go too well, that I couldn't really comprehend what he was saying*.

Common to this and several other stories related by parents is that the paramedics stepped out of their strict professional roles and displayed care and empathy in a fundamental and personal way. This communicative care was not fully grounded in narrow rules and procedures, and it had a significant positive emotional effect on the parents. The mother who received the wink mentioned above said that the experience contributed to 'helping me for many years'. All of the families who experienced empathy and care in some way appreciated it enormously.

The above stories also illustrate how single actions and details that might seem unimportant were often hugely meaningful for the parents. Sometimes something as simple as a paramedic's tone of voice was perceived as important. One of the parents referred to the sentence 'We are doing as well as we can', referring to resuscitation. This statement was uttered in a 'way that made it clear they did not have very high expectations'. Nevertheless, the parents emphasised that it was uttered in a way that expressed care and acknowledgement of the parents' perspectives.

We found that one family experienced little or no empathy at all. The mother in this family felt that she was 'being ordered to do various things', and that she was not allowed to make her own decisions. She felt that she was not taken care of properly. For example, when her child was taken to the hospital, the mother was transported in a separate ambulance. We were also told a story about siblings of a lifeless child who were placed in a separate room. The parents thought that the paramedics' motivation for this was that the other children should not see and interfere with their resuscitation. The negative consequence of this arrangement was that the siblings were later very angry with the 'men in uniform' and thought that they 'had done something bad to the baby'. However, while a majority of the parents had some critical comments relating to the paramedics' communication of empathy, only one family reported episodes that they thought involved 'a total lack of empathy'.

Most of the families were reasonably satisfied with the practical arrangements that were made during the encounters, but some of our respondents pointed to minor negative episodes. Typical examples of such episodes included transportation arrangements and accompaniment to the hospital. One parent held that 'They should have guided us better into the continuing system'. Another family found a mess from the resuscitation activities when they returned from the hospital: 'They could have cleaned up better before they left'. However, this was not presented as a strong criticism, and the parents made it clear that they understood that 'It is difficult to take care of all the practical matters in a situation like this'. From the perspective of the parents, criticism was only justified when it was reasonable to expect more from the paramedics than what they actually did. The parents who were critical of the disorder in their house were critical because many paramedics and relatives were present at the time: 'It should have been possible for one ambulance to remain at our house for a while.'

Another factor conceived to be significant was the time constraint. One family experienced empathy and care related to this. In this case the paramedics had given up resuscitation and 'gave us time to attend to and dress our baby'. This was something the parents appreciated a great deal. Some less attentive paramedics, perhaps, would not have given the parents this opportunity to spend time with their child.

What then about the paramedics involved in this kind of interaction? Do they agree that their communication and care could be better? Perhaps the most striking result is that the paramedics have very different views about what the parents are entitled to expect. On the one hand, there is the view is that it is overwhelmingly difficult for paramedics to secure good communication with the parents and give them substantial care:

*It's hard to act naturally when children are involved. We could do. The reason it doesn't happen is that we can't be professional in that role. We are really very reluctant to do it. It's easier to focus on the technical things, and we absolutely dread turning round and looking them straight in the face*.

This view, however, is not shared by all paramedics, who may instead share this opinion:

*I completely disagree that requiring good communication skills is asking too much. I find that (opinion) fairly provoking. Eighty percent of our job is to show consideration whether you're out on an emergency or on routine duty. All many patients want is a hand to hold, somebody who is there for them*...

Why do the paramedics disagree to such an extent? One of the reasons might be the fact that most of the paramedics expressed scepticism about the parents' ability to communicate. As one said, 'People in a state like this are not able to communicate very much'. One of the paramedics supported this view with a personal experience. His own child became very ill, and he experienced how difficult it was to do even the most straightforward practical tasks, such as dialling for emergency assistance. What he wanted when other paramedics arrived was for them to focus on the child. He himself was in a state of shock and found it very difficult to communicate with anyone.

Most of the paramedics also had a clear impression that the parents wanted them to focus on the lifeless child. As one characteristically said: 'When we arrived we ran past the mother and into the room where the child was'. In another case, the paramedic expressed that 'We heard the mother scream from a room inside the house and hurried into the room to start resuscitation'. Several of the paramedics emphasised that since it is so challenging to perform resuscitation on a small child, it is difficult to communicate and interact with the parents. Others, however, maintained that communication with the parents should happen as long as there are no practical obstacles to it. All of the paramedics agreed that the context of the situation is crucial. As one said, 'This [communication with the parents] depends a lot on the situation. If many [paramedics] are present, then it is easier. If there are few present, then it is much more difficult.'

## 6. Discussion

There is significant disagreement among the paramedics interviewed about their professional role as caregivers. A possible explanation of this disagreement is that the paramedics have different personalities and backgrounds. However, our findings show that the paramedics do not think that their opinion merely reflects their personality or 'character'. When they speak about what people in crisis are entitled to expect from them, they tend to speak about what they are entitled to expect in general.

Determining how paramedics should communicate and offer care in emergency situations involving persons in crisis is an issue that must be confronted on a systematic level. In our opinion, guidelines that specify common threshold conditions for communication and care that all paramedics can meet should be developed and implemented in large-scale education and training efforts. A challenge that confronts a project of this kind is that comprehensive guidelines have to be based on theoretical assumptions about personal crises and the human mind. Different psychological perspectives will recommend different actions and strategies in interaction with people in crisis [1,7]. The question that arises is this: What kind of theoretical perspective should form the basis for paramedics' actions towards patients in crises? In order to develop valid, substantial guidelines, this is a question that needs to be addressed.

Meanwhile, we propose that it is possible to state some guidelines that are less dependent on psychological theories. As illustrated above, our research suggests that it is often 'the small things' such as a wink of friendly gesture that matter to parents involved in crisis situations. This observation was especially salient in connection with empathy. This observation, combined with the fact that all of the parents felt that communication of explanations and information could have been better, suggests that paramedics should always be aware of the level of care, communication and empathy provided to patients and persons in crisis.

If it were necessary for the paramedics to have comprehensive theoretical knowledge of the human mind and communication processes in order to achieve successful communication with the parents, then it would be unrealistic to demand they attempt it at all time. But as one of our informants so aptly says, 'It takes so little to show a minimum of empathy and care'. We have given several examples of this above, and we think it is reasonable to conclude that paramedics and other health care professional members should be able to meet such minimalist expectations when the situation makes it possible.

Obviously, it is impossible to give any kind of general instruction for doing all the 'small things' that patients and relatives of patients tend to appreciate so much. Each situation and the perspectives of the persons involved differ, so guidelines will always have to be interpreted and adjusted accordingly. Furthermore, while possessing basic empathy is crucial to avoiding poor communication and substandard care, empathy cannot be reduced to specifications of action-guiding rules.

Even though rigid rules of communication for interaction between emergency personnel and parents of lifeless children cannot be developed, we think it should be possible to develop some 'soft guidelines' within the scope of the kind of situations covered by our study. We suggest that the aim of developing such guidelines should be that all parents of lifeless children are secured a minimum of relevant explanations, information and care. Obviously, such guidelines will also be relevant in other cases of interaction involving persons in crisis. To this end, our research indicates that the actions of paramedics' in a range of cases are not always carried out in accordance with reasonable minimum expectations. We therefore think that 'soft-guidelines' can play an important role in a variety of cases, and that attentive paramedics, in their daily interactions with patients and relatives of patients, are in the best position to understand how the guidelines would apply to specific situations.

## Authors' contributions

HN participated in the design of the study, analysed the data and drafted the manuscript. ØN conceived of the study and participated in its design and coordination. All authors read and approved the final manuscript.
